# Human Immunodeficiency Virus and Pulmonary Arterial Hypertension

**DOI:** 10.1155/2013/903454

**Published:** 2013-08-21

**Authors:** Aibek E. Mirrakhimov, Alaa M. Ali, Aram Barbaryan, Suartcha Prueksaritanond

**Affiliations:** Department of Internal Medicine, Saint Joseph Hospital, 2900 North Lake Shore, Chicago, LL 60657, USA

## Abstract

Human immunodeficiency virus- (HIV-) related pulmonary arterial hypertension (PAH) is a rare complication of HIV infection. The pathophysiology of HIV-related PAH is complex, with viral proteins seeming to play the major role. However, other factors, such as coinfection with other microorganisms and HIV-related systemic inflammation, might also contribute. The clinical presentation of HIV-related PAH and diagnosis is similar to other forms of pulmonary hypertension. Both PAH-specific therapies and HAART are important in HIV-related PAH management. Future studies investigating the pathogenesis are needed to discover new therapeutic targets and treatments.

## 1. Introduction

Pulmonary hypertension (PH) is a group of disorders characterized by a mean pulmonary arterial pressure (mPAP) ≥25 mmHg during right heart catheterization [1]. The most recent PH classification includes five main groups, which are shown in Table 1. The disease is called pulmonary arterial hypertension (PAH) when classified as group 1 PH and is called PH when the etiologic factor fits into other groups (groups 2–5). The association of PH with other medical disorders generally imparts a worse prognosis. For example, the presence of PH in patients with chronic obstructive pulmonary disease (COPD) is linked to greater morbidity and mortality [2]. More detailed discussion of PH is beyond the scope of this paper and can be found elsewhere [3, 4].

Human immunodeficiency virus (HIV), which belongs to the lentivirus subgroup of the retrovirus family, became known after describing cases of pneumocystis pneumonia and Kaposi sarcoma in 1981 [5, 6]. It is believed that at least 35 million people are infected with HIV worldwide [7]. HIV infection, if left untreated, can lead to a profound decline in host immunological function which in turn predisposes to a myriad of infectious diseases [8, 9] and malignant conditions [10]. Furthermore, recent evidence suggests that patients with HIV are at greater risk of cardiovascular disease and kidney disease, even after adjustment for conventional risk factors [11, 12]. Further discussion of HIV-related topics is far beyond the scope of this manuscript and can be easily found elsewhere. 

The goal of this paper is to summarize the current knowledge on HIV-related PAH, which belongs to group 1 PH (see Table 1). First, we will discuss the epidemiology of HIV-related PAH. Second, clinical features of PAH will be reviewed. Third, studies assessing prognostic factors and HIV-related PAH morbidity and mortality will be reviewed. Fourth, the current understanding of the HIV-related PAH pathogenesis will be covered. Fifth, clinical studies and current treatment options for patients with HIV-related PAH will be mentioned. 

## 2. Epidemiology of Pulmonary Hypertension in Patients with Human Immunodeficiency Virus

HIV-related PAH was first described in 1987 by Kim and Factor, who presented a 40-year-old male with membranoproliferative glomerulonephritis and plexogenic pulmonary vasculopathy [13]. In 1991, Speich et al. studied 74 HIV patients (out of 1,200 HIV patients in the cohort) with respiratory complaints [14]. Doppler echocardiography, electrocardiograms, chest roentgenography, and ventilation perfusion scans were used during the study. These researchers demonstrated that the incidence of HIV-related PAH was about 0.5%, which is several times greater than in the general population [15]. Sitbon et al. analyzed the cohort of 7,648 HIV patients in France [16]; 247 patients with HIV who had respiratory complaints and no prior history of PH underwent echocardiography and right cardiac catheterization. These researchers reported 0.45% prevalence of HIV-related PAH, which is close to the prevalence reported by Speich et al. [14]. 

Mehta et al. analyzed the published clinical literature to date (published in 2000) regarding HIV-related PAH [17]. These investigators reviewed 131 published cases of HIV-related PAH and showed that 53% of patients were males, mean age at diagnosis was 33 years, and interval between HIV diagnosis and the diagnosis of HIV-related PAH was 33 months. Shortness of breath, pedal edema, cough, fatigue, syncope, and chest pain were the most common presenting signs of HIV-related PAH. Right ventricular hypertrophy was the most common finding on the electrocardiogram, and right cardiac dilation was the most frequent observation on echocardiography. It was shown that plexogenic pulmonary vasculopathy was the predominant finding on autopsy, which is similar to pathological findings of idiopathic PAH.

Humbert et al. studied 674 patients with PH to assess the etiology and risk factors [18]. They found that HIV-related PAH was responsible for about 6.2% of cases. Quezada et al. studied 392 HIV patients to investigate the risk factors for HIV-related PAH [19]. It was demonstrated that the prevalence of HIV-related PAH was 9.9% based on the echocardiographic assessment. After performing multivariate logistic regression analysis, it was shown that female gender and detectable HIV viral load were associated with the presence of PAH. 

It is essential to mention that some reports did link intravenous drug use (IDU) as an independent risk factor for HIV-related PAH since the majority of patients in this cohort were injection drug users [20]; however, others failed to demonstrate such association [21]. On the top of that, intravenous drug users with HIV-related PAH were not clinically different to other patients with HIV-related PAH [22].

Reinsch et al. studied 802 HIV-infected patients to assess the impact of antiretroviral treatment on HIV-related PAH [23]. These researchers demonstrated that patients on antiretroviral therapy and female patients had a greater prevalence of HIV-related PAH based on echocardiography findings. They also mentioned that the prevalence of HIV-related PAH is underestimated since clinical studies tend to include symptomatic patients. Isasti et al. confirmed the notion regarding underestimation of the prevalence of PH in a study of 196 HIV-infected individuals who lacked respiratory or cardiac complaints [24, 25]. It was demonstrated that 2.6% of patients did have evidence of PH and 6.1% had right ventricular systolic dysfunction based on echocardiography. It is important to note that the prevalence reported in the study by Isasti et al. is several times greater than that in previous reports. Small study sample size, study methodology, and patients' demographics might account for such differences. Niakara et al., from Burkina Faso, also reported a greater than previously reported prevalence of HIV-related PAH in a study of 79 patients [26]. It was demonstrated that 5% of patients were found to have evidence of PH based on the noninvasive assessment. 

Morris et al. investigated 116 subjects with HIV infection [27]. Echocardiography, pulmonary function tests, sputum cell count and differentials, markers of T-lymphocyte activation, and levels of NT probrain natriuretic peptide levels were measured. It was shown that 18 subjects with HIV had evidence of elevated pulmonary arterial pressure. Markers of inflammation were increased in patients with HIV and were related to the presence of elevated pulmonary arterial pressure.

On the other hand, it is important to keep in mind that echocardiographic assessment of pulmonary arterial pressure is far from perfect, which is true for both HIV-infected individuals and patients without HIV infection. For example, Selby et al. reported that echocardiographic assessment of pulmonary arterial pressure was inaccurate in 19.7% of patients compared to right heart catheterization [28]. Based on the published reports, it is clear that patients with HIV-related PAH have worse outcomes compared to HIV patients without PAH (this will be discussed in more detail in the following sections). Also, it seems that HIV-related PAH may be diagnosed in patients with any degree of HIV-related immune deficiency (this will be discussed later in the text). 

In summary, the estimated prevalence of HIV-related PAH ranges from 0.4 to 5%. Various study populations and methodological differences may explain the various epidemiological findings. Female gender, injection drug use, and the use of antiretroviral medications may serve as risk tools for clinical use to determine patients at risk. However, it is essential to keep in mind that the finding regarding the association between use of antiretroviral medications and the risk of HIV-related PAH may be flawed, since the study was done in a developed country, where most patients generally have access to antiretroviral therapy. Another important consideration is that HIV-infected individuals tend to live longer in the era of highly active antiretroviral therapy (HAART) and, thus, may have a greater incidence of PH. It is also important to consider that patients with HIV infection may have a greater medical surveillance, and, thus, these patients may be diagnosed at early disease stages. 

## 3. Clinical Presentation of PH

Clinical presentation of PH is nonspecific, and patients typically present with exertional symptoms such as progressive dyspnea, chest pain/discomfort, and in advanced cases dizziness and syncope [1]. Cough, hemoptysis, fatigue, and lower extremity pitting edema are also common presenting features. These symptoms are nonspecific, of course, and overlap with those of many other diseases, including pulmonary and cardiac conditions. Physical examination can be very helpful in signaling problems in the pulmonary circulation. An early manifestation can be an increase in the intensity of the second pulmonic sound (P2), but as the disease progresses, the right ventricle enlarges so that an impulse or heave can be appreciated in the left lower sternal border. The tricuspid valve becomes more incompetent as the RV enlarges, so that a holosystolic murmur indicative of tricuspid regurgitation becomes audible in the left and right lower sternal borders. The onset of RV failure leads to jugular venous stenosis, hepatojugular reflux, peripheral edema, and ascites. These are not specific for isolated right ventricular failure and are commonly seen with left ventricular dysfunction as well.

During physical examination, it is essential to search for signs of systemic autoimmune diseases (particularly systemic sclerosis) such as joint pains, skin thickening, malar rash, telangiectasia, and Raynaud's phenomenon [29]. 

## 4. Laboratory and Instrumental Workup in the Diagnosis of PH

It is important to consider the diagnosis of PH in patients with shortness of breath, syncope, fatigue peripheral edema, and abdominal discomfort. The symptoms and signs mentioned above are nonspecific for PH. Physical examination is essential and should focus on the signs presented in the previous section. 

Several instrumental tests can be of help in evaluating patients with the aforementioned complaints and physical signs. 12-lead electrocardiogram (ECG) is a relatively inexpensive diagnostic tool, which can also assist in detecting other potential explanations for the patients' presentation. ECG may reveal “p” pulmonale consisting of tall P waves (≥3 mm) in the inferior leads, right axis deviation, or right bundle branch block and R to S ratio > 1 in lead V1, indicative of right ventricular hypertrophy. ST depressions in leads V1 to V3 suggest right ventricular strain in combination with other findings. However, these findings have limited sensitivity for diagnosing PH, especially in patients with mild PH, and are of limited prognostic value [30].

Chest X-ray (CXR) is an important test for evaluating patients presenting with shortness of breath, chest pain, and other respiratory-related symptoms, as PH is one of many conditions that can present with these symptoms (such as pulmonary hyperinflation secondary to chronic obstructive pulmonary disease). In PH, the CXR may be completely normal but is apt to show evidence of RV or PA enlargement in advanced cases. However, the utility of CXR in diagnosing PH is limited due to its lack of sensitivity.

Echocardiography in patients with PH may show an increase in pulmonary artery pressure, as well as right atrial and enlargement of the RV. It also provides important information on left heart function. Despite having much greater sensitivity and specificity for PH than the ECG and CXR, the cardiac echo may under- or overestimate the degree of pulmonary artery pressure [31], which is also true for patients with HIV-related PAH [28].

Pulmonary function tests (PFT) are important mostly to exclude other potential causes of PH (such as chronic obstructive pulmonary disease and interstitial lung diseases). However, patients with PAH may have mild to moderate decreases in forced vital capacity, forced expiratory volume in one second, and diffusion capacity for carbon monoxide compared to healthy control subjects [32, 33]. 

Nuclear ventilation perfusion (V/Q) scans are typically performed in order to exclude chronic thromboembolic pulmonary hypertension (CTEPH) [4]. V/Q scans can show large ventilation perfusion mismatch [34] but may underestimate the severity of CTEPH [35], especially central clot load. Other limitations of V/Q scans are decreased sensitivity and specificity in patients with underlying pulmonary disease and low specificity, since other pathologies may give similar findings. Due to the limited specificity of V/Q scans, patients with positive findings typically undergo further imaging studies to better quantify disease severity and exclude alternative diagnoses. As long as renal function is acceptable, computed tomography-pulmonary angiography (CT-PA) is usually performed next in cases of suggestive V/Q scans, since it has greater specificity than V/Q scans and can help exclude other diseases. 

Pulmonary angiography (PA) and right heart catheterization is the mainstay of diagnosis confirmation and disease prognostication. Measurement of mPAP at rest and mean pulmonary capillary wedge pressure (mPCWP) is performed during right heart catheterization. PH is diagnosed when mPAP during rest ≥25 mmHg and mPCWP < 15 mmHg. The measurement of mPCWP is important since PH secondary to left heart disease is essentially ruled out with value less 15 mmHg [1]. However, mPCWP > 15 may be secondary to dilation of pulmonary arteries. In such cases, it is essential to measure left ventricular end diastolic pressure. 

Several laboratory tests may be needed to better understand the etiology of PH in a particular patient. For example, in a patient who has risk factors for sexually transmitted disease it is important to screen for HIV infection. Vazquez et al. demonstrated that among 445 patients with PH, only 1 was found to be positive for HIV [36]. This patient had risk factors for HIV infection. Other tests which should be considered are antinuclear antibody and rheumatoid factor to screen for possible underlying systemic connective tissue disorder. Liver function tests, albumin level, and international normalized ratio may be useful in screening for portopulmonary hypertension in appropriate clinical scenario. Sickle cell disease is another important differential to consider which may lead to PH [37].

In summary, it is essential to exclude PH secondary to left-sided cardiac disease, PH secondary to pulmonary disease, CTEPH, and other etiologies in appropriate clinical scenarios (such as portopulmonary hypertension and PH secondary to sickle cell disease) before attributing the etiology to PAH.

## 5. Prognostic Factors and Mortality in Patients with HIV-Related PAH

It is essential to state that patients with HIV-related PAH have a worse prognosis compared to subjects with HIV alone. This was demonstrated in 1997 in a study by Opravil et al. from the University of Zurich, Switzerland [38]. These researchers enrolled 19 patients with HIV-related PAH and 19 control subjects with HIV infection alone. It is essential to note that antiretroviral and PAH-focused therapy was not widely available at that time. Nevertheless, it was demonstrated that patients with HIV-related PAH had a worse survival (approximately two times) compared to patients without HIV-related PAH. The authors showed that the diagnosis of HIV-related PAH and lower CD4 cell count were associated with decreased survival. However, it is important to keep in mind that the study was performed when antiretroviral therapy was not widely available, and the association between CD4 cell count and mortality may simply portray a greater risk for opportunistic infections and diseases associated with profound immunosuppression. 

Nunes et al. studied 82 patients with HIV-related PAH to investigate the prognostic factors for this disorder [20]. The authors demonstrated that greater New York Heart Association (NYHA) functional class at the time of diagnosis was associated with a poorer survival. After performing multivariate analysis, CD4 cell count >212 cells mm^−3^ was associated with a better prognosis. Epoprostenol infusion and antiretroviral therapy were not independently associated with improved survival.

Degano et al. investigated the prognostic factors for HIV-related PAH in 77 patients [22]. All patients were receiving antiretroviral therapy, and 50 subjects were started on PAH-specific therapies. Antiretroviral therapy led to an improvement in physical endurance, but not in hemodynamic parameters. PAH-specific therapies were found to improve both hemodynamic parameters and physical endurance. It was demonstrated that lower CD4 cell count and cardiac index <2.8 L/min/m^2^ were associated with a worse prognosis. 

Furthermore, it is important to mention that patients with HIV-related PAH have a similar prognosis compared to patients with idiopathic PAH [39].

Based on the current data, worse functional class, lower cardiac index, and lower CD4 cell count are predictors of worse outcomes among patients with HIV-related PAH.

## 6. Pathogenesis of HIV-Related PAH

The pathogenesis of HIV-related PAH is complex and not entirely understood. The pathogenesis model may be subdivided into several aspects with the purpose of better understanding the problem. However, it is essential to note that many contributing factors play a simultaneous role in the pathogenesis of HIV-related PAH. It is important to state that on a histological level HIV-related PAH is similar to other causes of PAH [17].

First, as described previously, patients with HIV have a greater incidence of PAH compared to the general population [15]. Therefore, based on the epidemiology data it is prudent to conclude that HIV infection has some effects (either direct or indirect) on pulmonary vasculature. It is interesting to note that current scientific data suggest that HIV does not directly infect pulmonary endothelial cells [40, 41]. However, HIV contains several proteins which are implicated in the pathogenesis of HIV-related PAH that can participate in the pathogenesis of PAH. One such HIV-related factor is the negative factor or Nef, which is essential for HIV replication and pathogenesis [42]. It was shown in a macaque model that Nef-positive simian immunodeficiency virus (SIV) induces plexiform pathology in the pulmonary vasculature compared to SIV negative for Nef [43]. It was shown that Nef can enter into the pulmonary endothelial cells via the CXCR 4 receptor [44] and can induce proliferation and apoptosis (programmed cell death) of pulmonary endothelial cells [44].

Tat is another HIV-related protein that has been shown to enhance the activity of vascular endothelial cells via interleukin-6 [45]. More recently, the Tat protein was shown to repress the transcription of bone morphogenic protein receptor 2 (BMPR-2) and led to abnormal pulmonary vascular function with exuberant cellular proliferation [46]. It is relevant to mention that BMPR-2 is among the most studied factors implicated in the pathogenesis of PAH [47].

Glycoprotein 120 (Gp-120) is a crucial protein responsible for HIV entry into the target cells, such as macrophages and CD4 T lymphocytes [48]. It was shown that Gp-120 induces vascular smooth cell proliferation and endothelial cell apoptosis as well as increases the production of endothelin-1, which is a well-established culprit molecule in the pathogenesis of PAH [41, 49]. 

Nevertheless, it is essential to state that only a small proportion of infected patients with HIV will develop HIV-related PAH. Other factors, including genetic susceptibility, likely play a role in the pathogenesis of this type of PAH. It was shown that platelet-derived growth factor is overexpressed in patients with HIV-related PAH [40]. However, a recent study did not identify any mutations in BMRP-2 among HIV-infected patients with PAH [20].

Mermis et al. showed in a rat model that HIV-related proteins (Gp-120 and Tat) led to oxidative stress with the resultant activation of hypoxia-inducible factor-1 and platelet-derived growth factor [50]. The authors speculated that this might be a pathological pathway implicated in the occurrence of HIV-related PAH. 

HIV infection is associated with enhanced systemic inflammation [51]. It is believed that HIV-related inflammation is among the key factors responsible for a greater burden of cardiovascular disease among HIV-infected patients [11]. However, it is essential to consider conventional cardiac risk factors since they contribute the most to the occurrence of cardiovascular disease [52]. 

HIV-related systemic inflammation may activate platelet derived growth factor [39] and vascular endothelial growth factor pathway [53], with resultant aberrant pulmonary vascular activity.

Certain viral infections were implicated in the pathogenesis of PAH. Human herpes virus 8 (HHV-8) is a DNA virus implicated in the pathogenesis of the Kaposi sarcoma, the Castleman disease, and primary effusion lymphoma [54]. Cool et al. demonstrated that HHV-8 related latency nuclear antigen 1 was present in 62% of lung tissue samples from patients with sporadic PAH, but not in lung tissues of secondary PH [55]. HHV-8 is common among HIV-infected homosexual men [56], and male to male sexual intercourse is the most common route of HIV acquisition in the USA [57]. However, other research groups failed to identify HHV-8 as a risk factor for PAH [21, 58, 59]. Other viral infections such as hepatitis B virus, hepatitis C virus, and cytomegalovirus were hypothesized to play a role in the pathophysiology of PAH [60]. It is interesting to note that previous Pneumocystis infection was shown to lead to PH via deranged immune response to the pathogen [61].

IDU is a common route for the acquisition of HIV and other infectious diseases. For example, most of the patients in the study by Nunes et al. were intravenous drug users [20]. Dhillon et al. showed that cocaine use had additive effects to HIV infection on the pulmonary vascular endothelial dysfunction and smooth muscle cell proliferation [62]. Activation of platelet-derived growth factor was implicated in the pathobiology of the above findings. Spikes et al. showed that morphine injection had additive effects to the simian immunodeficiency virus on the occurrence of PAH in a macaque model of HIV-related PAH [63]. Inflammation involving the pulmonary vasculature with resultant aberrant cellular apoptosis and proliferation were hypothesized to underlie the pathophysiology of association between IUD and HIV infection [63, 64]. 

Other factors may also contribute to the burden of HIV-related PAH. For example, patients with HIV infection may have a greater burden of venous thromboembolic events compared to the general population [65]. Therefore, it is possible that chronic pulmonary embolization may lead to CTEPH-like pathology similar to other conditions (particularly anti-phospholipid antibody syndrome) [4]. It is interesting to note that Hebert et al. showed that indinavir and zidovudine combination led to enhanced synthesis of endothelin-1 via medication associated mitochondrial toxicity [66]. 

In addition, certain human leukocyte antigen (HLA) may be implicated in the pathogenesis of HIV-related PAH. Morse et al. showed that HLA-DR6 and HLA-DR52 were common in patients with HIV-related PAH [67]. The authors speculated that the HIV-related PAH in these individuals may have an autoimmune basis. It is also essential to mention that left-sided heart disease, which is common in patients with HIV, can also contribute to the elevated pulmonary arterial pressure.

In conclusion, the pathogenesis of HIV-related PAH is complex with many factors playing a role. Viral proteins and factors, HIV-related inflammation, IUD, coinfections with certain organisms, genetic susceptibility, and possible other factors contribute to the occurrence of HIV-related PAH. Therefore, future studies should further explore the pathobiology of HIV-related PAH, which can lead to discoveries of new therapeutic targets. 

**Figure 1:**
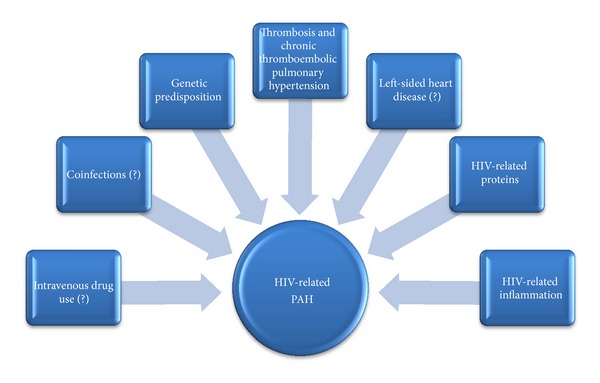
An overview of the pathogenesis of HIV-related PAH.

An overview of the pathogenesis of HIV related PAH is presented in Figure 1. 

## 7. Clinical Management of HIV-Related PAH

This section will be subdivided into several subsections for a better understanding of various options in the management of patients with HIV-related PAH. It is important to note that the comprehensive review of PAH management is beyond the scope of this paper and can be easily found elsewhere. 

### 7.1. Adjunctive Therapies

It is essential to note that the treatment of HIV-related PAH is similar to the treatment of other forms of PAH. Several medications may be adjunctively used in the management of PAH. First, patients with PAH are at increased risk of intrapulmonary vascular thrombosis due to prothrombotic state, sedentary lifestyle, and cardiac dilation. In such populations, even a small intrapulmonary thrombus can lead to acute right-sided heart failure and death [68]. Therefore, chronic therapy with anticoagulation should be instituted in patients with PAH, including HIV-related PAH. Among anticoagulants, the use of warfarin is recommended given the availability of scientific data in patients with PAH [68]. International normalized ratio (INR) should be periodically assessed in patients using warfarin with an INR goal of 1.5–2.5, which may be associated with a better survival in patients with PAH [68]. 

Patients with PAH often develop hypoxemia, which can further exacerbate pulmonary arterial vasoconstriction [68]. Such patients should be treated with supplemental oxygen. Another common complication of any form of PH is the development of right-sided heart dysfunction. Such patients are classically volume overloaded, and therapy with diuretic agents may be of benefit in terms of reduced liver congestion [68]. However, it is essential to note that diuretic therapy should be carefully monitored in patients with PAH, since these agents can decrease cardiac venous return and preload and lead to hypokalemia, which may lead to fatal heart arrhythmia in the settings of dilated heart and the development of metabolic alkalosis. The development of diuretic-associated metabolic alkalosis can lead to respiratory acidosis (via physiologic acid base compensatory mechanism), which can be dangerous to patients with PAH. 

Digoxin can be used in patients with PAH and right-sided heart failure to reduce symptoms. In addition, it may be useful in controlling the heart rate in PAH patients with concomitant supraventricular arrhythmias. However, it is important to note that the scientific basis for digoxin use in patients with PAH is scant [68].

Calcium channel blocker (CCB) is a heterogeneous class of medications consisting of various agents with different effects on cardiac conduction system. Amlodipine, diltiazem, and nifedipine are the most commonly used medications in the treatment of PAH [69]. It is important to note that prior to prescribing CCB, the vasoreactivity test needs to be performed [69]. The vasoreactivity test is performed with the administration of vasodilator agent (typically adenosine or epoprostenol or inhaled nitric oxide). CCB trial should be considered only in patients who develop decrease in mPAP of at least 10 mmHg and to a value less than 40 mmHg. This reduction in mPAP must be accompanied by improved or at least unchanged cardiac output and unchanged or minimally reduced systemic blood pressure [68]. Unfortunately, only a small minority of patients with PAH will be classified as having a positive vasoreactivity test. Patients who respond to CCB should be periodically followed and reassessed whenever appropriate. In patients who do not meet the above criteria, CCB therapy is not recommended.

In conclusion, it is essential to mention that there is a lack of data regarding the use of above medications in patients with HIV-related PAH. Nevertheless, it is believed that these medications and approaches may be clinically useful in appropriate clinical situations in patients with HIV-related PAH (e.g., patients with hypoxemia will benefit from supplemental oxygen). 

### 7.2. PAH-Specific Therapies

Several agents are available in the management of PAH, such as endothelin receptor antagonists, prostaglandin analogs, and phosphodiesterase 5 inhibitors (PDE-5) [68]. Endothelin receptor antagonists are orally administered medications, with bosentan, sitaxsentan, and ambrisentan being available on the market. Prostaglandin analog class contains parenterally administered medications such as epoprostenol and treprostinil as well as inhaled iloprost. Class of PDE-5 includes orally administered sildenafil, tadalafil, and vardenafil. 

It is important to note that classification of PAH severity by the World Health Organization (WHO) is paramount in deciding which therapy to use [70]. The WHO functional classification of PAH is presented in Table 2 (adapted from [70]). For example, patients with WHO functional classes II and III can be initially managed with any oral agents unless contraindicated. Patients with WHO functional class IV should be started with prostaglandin analogs plus the addition of other medications if needed [68].

Later, we will review the clinical data on the use of PAH-specific therapies in settings of HIV-related PAH. 

PDE-5 inhibitors block the cellular degradation of cyclic guanosine monophosphate, thereby leading to vasodilation. Several case reports were published regarding the use of sildenafil in patients with HIV-related PAH [71–73]. These case reports showed beneficial effects of sildenafil on patients' symptomatology and hemodynamic parameters. However, it is important to keep in mind the potential interactions with protease inhibitors (which are part of antiretroviral therapy) such as ritonavir and indinavir, which can increase sildenafil concentration [74]. However, Chinello et al. did not show any increase in adverse effects, despite the higher concentration of sildenafil in two patients with HIV-related PAH [75]. Therefore, it may be prudent to use a lower sildenafil dose in patients using protease inhibitors. To our knowledge, there are no reports regarding the use of other PDE-5 inhibitors in patients with HIV-related PAH.

Endothelin receptor blockers are an important part of PAH treatment. Sitbon et al. investigated the utility of bosentan in 16 patients with HIV-related PAH [76]. This study showed that bosentan therapy for 16 weeks led to an improvement in exercise capacity, quality of life, and hemodynamics as well as echocardiography variables. It is important to note that no issues regarding hepatotoxicity of bosentan were noted among patients with HIV-related PAH (this is of concern given that HIV patients are at increased risk for hepatotoxicity and bosentan is associated with abnormalities in liver function tests). Degano et al. studied the utility of bosentan therapy in 59 patients with HIV-related PAH [77]. Bosentan therapy was shown to be beneficial in terms of symptomatology, exercise capacity, and hemodynamics. It is important to note that both studies did not show any evidence that bosentan therapy led to worse HIV control [76, 77].

Several reports highlighted the beneficial effects of prostaglandin-based therapy of HIV-related PAH. Aguilar and Farber studied 6 patients with HIV-related PAH to assess the impact of epoprostenol therapy [78]. Follow-up cardiac catheterization showed improved hemodynamic parameters, and patients experienced a better quality of life with improvement in NYHA functional class. Cea-Calvo et al. showed improvements in 6-minute walk test and NYHA functional class in 3 patients with HIV-related PAH treated with treprostinil [79]. Ghofrani et al. enrolled 6 patients with severe HIV-related PAH to investigate the utility of inhaled iloprost [80]. These researchers showed that iloprost was associated with an improvement in the 6-minute walk test and pulmonary vascular function. 

A very interesting report was published in the European Respiratory Journal in 2012 [81]. The authors presented two patients in whom bosentan was successfully discontinued and no PAH recurrence was noted after 4 years of followup. The authors suggested that HIV-related PAH-specific therapy might be discontinued if the patients fulfill at least two criteria: hemodynamic normalization for at least 1 year and fully controlled HIV disease. However, their results should be replicated in larger studies prior to making any recommendations regarding the possibility of successful cessation of PAH therapies. 

In conclusion, it is relevant to note that all patients with PAH should be closely followed. Functional assessment with the 6 minute walk test is important to assess the functional status of the patients [68]. Small studies of PAH-specific therapies in patients with HIV are encouraging; however, studies with a large sample size are desired to provide a robust data regarding the management of HIV-related PAH. Nevertheless, until more specific data are available, patients with HIV-related PAH should be managed in a similar manner compared to other forms of PAH. 

### 7.3. The Role of Highly Active Antiretroviral Therapy in the Management of HIV-Related PAH

It is well-known fact that antiretroviral therapy is beneficial and its availability greatly reduced HIV-related morbidity and mortality [82]. Furthermore, it was shown that low CD4 cell count was predictive of poor outcome in patients with HIV-related PAH [22]. The same study showed that antiretroviral therapy was associated with improved 6-minute walk test; however, no improvement in hemodynamics was noted [22]. Furthermore, Zuber et al. in a retrospective study including 35 patients showed that HAART was related to improvement in right atrial pressure gradient assessed by echocardiogram as well as reduced from HIV-related PAH and other causes [83]. However, a prospective study performed by Nunes et al. showed that survival was worse in patients who were started on HAART in the absence of therapy with epoprostenol [20].

HAART should be initiated in all patients once the diagnosis of HIV is made [84]. HAART should not be used as a sole therapy for HIV-related PAH, and the initiation of PAH-specific therapy is of paramount importance. HAART can decrease the rate of infections in patients with HIV and thus, improve mortality in patients with HIV-related PAH. Pulmonary infections, including Pneumocystis infection, can exacerbate PAH and even lead to death in patients with preexistent pulmonary vascular disease. It is interesting to add that HAART may also mitigate hypoxia-related pulmonary vasoconstriction [85].

In conclusion, all patients with HIV-related PAH should be on HAART and PAH-specific therapy. Consultation with HIV specialist and pharmacist experienced in managing HIV patients may be required to minimize the risk of serious drug interactions. 

### 7.4. Surgical Options in the Management of PAH

Creation of a right to left shunt can be used in patients with advanced PAH in whom pharmacologic therapy is unable to control the disease. Atrial septostomy is one of the options which can be considered in patients with advanced PAH [86]. An opening between the right and left atriums is surgically created to bypass some of the blood from right atrium to the left atrium. Atrial septostomy should be considered in patients with advanced PAH and evidence of right-sided heart failure and frequent syncope in whom pharmacological therapy including diuretics is not effective. However, it is essential to note that patients with elevated mPAP, older patients, and patients with renal dysfunction and low cardiac output have worse outcomes after atrial septostomy. 

Another option is the creation of a shunt between left pulmonary artery and descending aorta which is known as a Potts shunt [87]. However, the perioperative mortality is high in adults and currently cannot be recommended for adult patients with PAH.

Lung and heart transplantation are reserved for patients with advanced PAH who do not respond to the above measures [88]. However, the presence of HIV was a contraindication to transplantation because of concerns regarding the immunosuppressive therapies in patients with HIV. However, recent reports suggest that patients with HIV infection can successfully undergo lung and heart transplantation with no adverse control of HIV infection [89]. 

In conclusion, it is important to note that the data on surgical management of PAH including transplantation lacks reports from patients with HIV-related PAH. Therefore, care of patients should be individualized, and selected patients should be referred to tertiary medical centers for further management.

## 8. Conclusion

HIV-related PAH is a rare complication of HIV infection. The pathophysiology of HIV-related PAH is complex, with viral proteins seeming to play the major role. However, other factors, such as coinfection with other microorganisms and HIV-related systemic inflammation, might also contribute. The clinical presentation of HIV-related PAH and diagnosis is similar to other forms of PH. Both PAH-specific therapies and HAART are important in HIV-related PAH management. Future studies investigating the pathogenesis are needed to discover new therapeutic targets and treatments. 

## Figures and Tables

**Table 1:** Classification of PH.

Group	Etiologies
Group 1.	Idiopathic, heritable, connective tissue disease, Human immunodeficiency virus infection, portopulmonary hypertension, congenital heart disease, drug/toxin induced, chronic hemolytic anemia, schistosomiasis, persistent PH of the newborn, pulmonary veno-occlusive disease and pulmonary capillary hemangiomatosis.

Group 2. PH owing to left heart disease	Systolic and diastolic dysfunction, mitral and aortic valve diseases.

Group 3. Pulmonary hypertension owing to lung diseases and/or hypoxia	Chronic obstructive pulmonary disease, interstitial lung disease, sleep disordered breathing, and so forth.

Group 4. Chronic thromboembolic pulmonary hypertension	Unresolved fibrin thromboembolization to the pulmonary arteries.

Group 5. Pulmonary hypertension with unclear multifactorial mechanisms	Myeloproliferative disorders, splenectomy, pulmonary vasculitis, neurofibromatosis, thyroid disorders, and so forth.

**Table 2:** The WHO functional assessment classification of PAH (adapted from [71]).

Class I:	Patients with PH but without resulting limitation of physical activity. Ordinary physical activity does not cause undue dyspnea or fatigue, chest pain, or near syncope.

Class II:	Patients with PH resulting in slight limitation of physical activity. They are comfortable at rest. Ordinary physical activity causes undue dyspnea or fatigue, chest pain, or near syncope.

Class III:	Patients with PH resulting in marked limitation of physical activity. They are comfortable at rest. Less than ordinary activity causes undue dyspnea or fatigue, chest pain, or near syncope.

Class IV:	Patients with PH with inability to carry out any physical activity without symptoms. These patients manifest signs of right-heart failure. Dyspnea and/or fatigue may even be present at rest. Discomfort is increased by any physical activity.
